# Comparison of the Effectiveness of the Miller Laryngoscope and the McGrath-MAC Video Laryngoscope in Direct Visualization of the Glottic Opening

**DOI:** 10.3390/medicina60010062

**Published:** 2023-12-28

**Authors:** Gamze Küçükosman, Keziban Bollucuoğlu, Mahmut Ava, Hilal Ayoğlu

**Affiliations:** 1 Department of Anesthesiology and Reanimation, Trabzon Faculty of Medicine, University of Health Sciences, Trabzon 61080, Turkey; 2 Department of Anesthesiology and Reanimation, Zonguldak Bülent Ecevit University, Zonguldak 67100, Turkey; keziban.bollucuoglu@beun.edu.tr (K.B.); mahmut.ava@beun.edu.tr (M.A.);

**Keywords:** miller blade, McGrath MAC videolaryngoscope, percentage of glottic opening, glottic view, direct laryngoscopy, lifting of epiglottis

## Abstract

*Background and Objective*: Placing the laryngoscope blade directly under the epiglottis (known as the direct view (DV) method) during videolaryngoscopy offers a superior view of the glottis when compared to the indirect method of lifting the epiglottis by positioning the Macintosh blade tip over the vallecula. While there are few studies comparing glottic views using Miller and Macintosh blades in pediatric patients, we have not come across such a study in adults. In this study, we aimed to compare the effectiveness and hemodynamic responses of the Miller laryngoscope and the McGrath-MAC videolaryngoscope (VL) in visualizing the glottic opening using the DV method. *Material and Methods*: A prospective study was conducted between August and December 2022 at XXX Hospital on 85 patients scheduled for surgical procedures involving endotracheal intubation. Patients were divided into two groups: Miller laryngoscope (Group M) and McGrath-MAC videolaryngoscope (Group VL) and intubated using the direct lifting method of the epiglottis. Hemodynamic responses before and after induction, as well as during laryngoscopy, intubation time, number of attempts, Cormack and Lehane (C&L) score, percentage of glottic opening (POGO), duration of the view of the opening, and need for external laryngeal pressure during intubation were recorded. *Results*: Both laryngoscopes showed similar effectiveness in terms of POGO and C&L score when used with the direct lifting method of the epiglottis. The median POGO values according to the DV method were 80% in Group M and 70% in Group VL (*p* = 0.099). Hemodynamic responses, intubation time, number of attempts, duration of view of the glottis opening, and the need for external laryngeal pressure were similar between the groups. *Conclusions*: Due to its ability to provide effective intubation conditions, we believe that the McGrath-MAC VL, when used with the indirect view method, can also be utilized in anesthesia practices alongside the DV method.

## 1. Introduction

The laryngoscope is the instrument used to visualize the larynx and intubate the trachea. The process of endotracheal intubation (ETI), performed to secure airway patency, is carried out using various laryngoscopes and methods. Laryngoscopes are classified as straight (Miller) or curved (Macintosh) based on the shape of their blade tips. During the process of ETI, direct laryngoscopes are used to align the oral, pharyngeal, and laryngeal axes and elevate the epiglottis, providing a view of the glottis. The most common method for this purpose is performed using the Macintosh laryngoscope, where the Macintosh blade tip is placed over the vallecula to indirectly (indirect view/IV) lift the epiglottis and expose the glottis. In the IV method performed with a curved blade laryngoscope, the laryngoscope is placed into the mouth from the right side of the mouth, pushing the tongue to the left. The tip of the blade is pushed up to the angle formed by the tongue and the epiglottis (vallecula), then lifted forward and upward. Thus, by removing the base of the tongue, the epiglottis and the structures in the mouth are removed from the field of view and a comfortable view of the glottis is provided. In laryngoscopy performed with a flat blade, after the epiglottis is seen, the blade is advanced to include the epiglottis. The laryngoscopy method performed in this way is a direct view (DV) method, and the epiglottis is held and lifted with the tip of the blade, making the glottis visible [1]. The Miller laryngoscope has been developed particularly for pediatric patients and challenging airway situations. It is known for providing a good view of laryngeal entry, effectively lifting the long and floppy epiglottis out of view during laryngoscopy by sliding the tongue to the left of the blade, which in turn improves the visualization of the glottic opening [2,3]. In recent years, a large number of laryngoscope instruments have revolutionized airway control using video technology [1]. The development of the videolaryngoscope (VL) is based on combining a conventional laryngoscope blade with an endoscopic system. This system integrates a camera with a specially designed handle to provide a clear and enlarged view of anatomical structures on a screen [1,4]. The advantages of the VL include the ability to perform laryngoscopy without aligning the axes of the oral cavity, pharynx, and larynx, and allowing ETI in patients with cervical spine anomalies and difficult airways. When using VL, there is no need to enter from the right side of the mouth. Blades can be placed from a midline approach, at a distance where the laryngeal structures are visible [1]. The McGrath-MAC VL (Medtronic, Watford, UK) is a recently developed Macintosh blade with a mounted monitor on the handle, designed for both direct and indirect laryngoscopy. The device is convenient for emergency situations due to the sterile single-use nature of the blades and its portability, making it readily available for use [1,5]. The effectiveness of this device in both normal and challenging airways has been reported in studies [5,6,7,8].

It has been reported that the use of the Macintosh blade is less stimulating compared to the Miller blade, as the former stimulates the glossopharyngeal nerve when inserted into the vallecula, while the latter lifts the epiglottis from the underside and stimulates the recurrent laryngeal nerve [9].

Recently, the intubation method has shifted from direct laryngoscopy to indirect laryngoscopy [7,8,9,10,11]. Most VL manufacturers recommend the IV method to lift the epiglottis. However, it has also been reported that using the DV method with the laryngoscope blade during VL can provide a better view of the glottis compared to the IV method [12]. Indirect laryngoscopy often allows visualization of laryngeal structures in difficult airways; however, imaging does not always guarantee successful ETT [1]. Except for the Miller blade, all other laryngoscope blades are known to lift the epiglottis and expose the glottic opening using both direct and indirect methods [6,7,8,9,10,11,12]. While there are few studies comparing laryngeal views using Miller and Macintosh blades in pediatric patients, we have not come across such a study in adult patients [9,13]. Our study primarily aims to compare the effectiveness of the Miller laryngoscope and the McGrath-MAC VL in visualizing the glottic opening using the DV method, and secondly to compare their hemodynamic responses.

## 2. Materials and Methods

### 2.1. Compliance with Ethical Standards

Our prospective study was conducted at Zonguldak Bülent Ecevit University Hospital, Zonguldak, Turkey, between August and December 2022, following approval from the local ethics committee (protocol No: 2022-14, ClinicalTrials.govIdentifier: NCT05820542) and obtaining informed patient consent.

### 2.2. Patient Population

The study included voluntary patients aged 18–65 years requiring ETI under elective conditions, undergoing non-cardiac surgery, and being within the American Society of Anesthesiologists (ASA) I–II risk group. Patients with allergies to the used medications, pregnant, those requiring rapid sequence intubation in emergencies, those with a history or suspicion of difficult intubation, morbidly obese patients, and those who did not provide informed consent were excluded from the study.

### 2.3. Application of General Anesthesia and Monitoring

**Figure 1:**
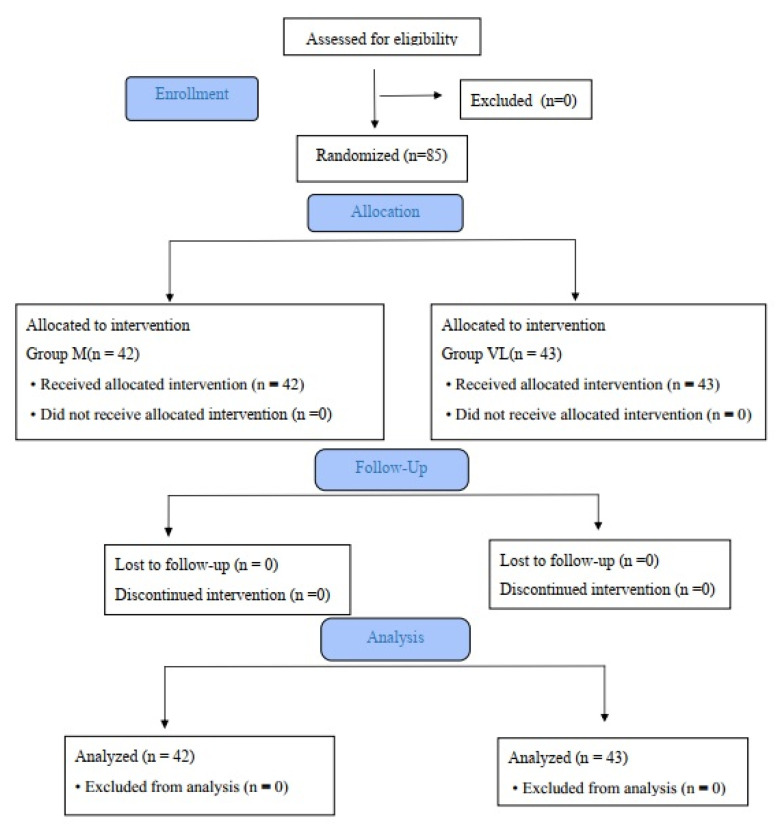
CONSORT flow diagram of the study.

The patients’ demographic characteristics and Mallampati and ASA risk scores were recorded. Non-invasive blood pressure monitoring, electrocardiograms, pulse oximetry (SpO_2_), bispectral index (BİS), and monitoring of neuromuscular blocking with a train of four (TOF ratio) were performed as standard monitoring in all patients. After preoxygenation, anesthesia induction was performed with fentanyl 1.5 mcg/kg, propofol 2 mg/kg, and rocuronium 0.6 mg/kg following the placement of a 7 cm high pillow under the patient’s head. Patients were randomized using a sealed envelope method into two groups: Miller laryngoscope (Group M) and McGrath-MAC VL (Group VL). Endotracheal intubations were performed by anesthesia assistants with at least 2 years of clinical experience, who had achieved a minimum of 30 successful intubations with both laryngoscopes. All laryngoscopies were performed with a BIS value between 40–60 and using a 3- or 4-numbered laryngoscope blade when TOF count was ‘zero’. Endotracheal tubes of size 7.0–7.5 for females and 7.5–8.0 for males were preferred. All intubation tubes were prepared with a stylet. The flow diagram according to CONSORT guidelines is provided in Figure 1 [14].

### 2.4. Data Management 

**Figure 2:**
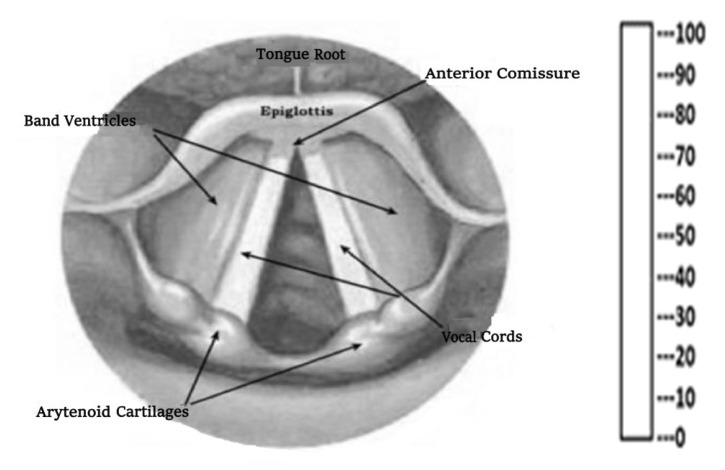
Percentage of glottic opening score.

Maneuvers and techniques facilitating improved glottic view, such as head positioning to enhance glottic view and external laryngeal pressure, were allowed and recorded. The Miller blade was placed through the right commissure into the mouth while the McGrath-MAC VL blade was placed along the midline of the tongue, and the shape and position of the epiglottis were confirmed [12]. Both blades were inserted into the mouth using the direct lifting technique for elevating the epiglottis, and subsequently, the percentage of glottic opening (POGO) and Cormack and Lehane (C&L) scores were evaluated. Visualizing the entire glottic opening, including the interarytenoid notch between the posterior cartilages of the vocal cords from the anterior commissure, corresponds to the POGO (100%) score. For the POGO score, a schematic representation with scores ranging from 0 to 100 was used to indicate the percentage of optimal glottic visualization during laryngoscopy (Figure 2) [15,16].

The practitioners were asked to mark the percentage of glottic opening on the line after each intubation. The optimal glottic view duration was defined as the time from when the practitioner placed the laryngoscope blade at the edge of the patient’s mouth to the moment they verbally confirmed viewing the glottis. C&L score (1 = most of glottis viewed, 2 = only posterior third of glottis viewed, 3 = glottis not viewed, only epiglottis viewed, and 4 = neither glottis nor epiglottis viewed) and intubation time were recorded, in addition to the glottic view duration, as the time elapsed from the beginning of glottic view to inflation of the endotracheal tube cuff. The number of attempts needed for successful ETI, hemodynamic parameters before induction (T1), after induction (T2), and during laryngoscopy (T3) were recorded. If the first intubation attempt was unsuccessful or terminated due to clinical reasons such as desaturation or the need for a position change, the laryngoscope was removed, and manual bag-mask ventilation was continued. A maximum of 3 intubation attempts were allowed, and participation was terminated if more than 3 attempts were required. Subsequently, any airway rescue device was used according to the clinician’s preference.

### 2.5. Statistical Analysis

The data were analyzed using the Statistical Package for the Social Sciences (SPSS) version 23.0 (SPSS, Inc. Chicago, IL, USA). When the results obtained from the ‘superiority of glottic view’ parameter in the reference article are used, a sample size of 38 cases per group was planned based on a power analysis using a Chi-square test with 95% confidence (1-α), 95% test power (1-β), and an effect size of w = 0.60 [12]. The normality of the data distribution was assessed using the Shapiro–Wilk test. Yates’ correction was used for the analysis of categorical variables based on group comparisons. The Mann–Whitney U test was employed for non-normally distributed data comparison between binary groups, and the independent two-sample *t*-test was used for normally distributed data comparison. Analysis results for quantitative data were presented as mean ± standard deviation (SD) or median (minimum–maximum) and for categorical data as frequency (percentage). The significance level was set at *p* < 0.050.

## 3. Results

### Clinical and Demographic Characteristics

Eighty-five patients were recruited for the study. Successful laryngoscopy and intubation procedures were carried out in all patients, and no patients were excluded from the study. There was no statistically significant difference between the groups in regards to patients’ characteristics and POGO score, glottic view, and intubation times, as shown in Table 1.

Most patients were successfully intubated on the first attempt. No significant differences were observed between the groups in terms of gender distribution, external laryngeal pressure requirement, ASA risk group, and intubation attempt count (Table 2).

No significant differences were found between the groups in terms of mean blood pressure, heart rate, and SpO_2_ at all measurement time points (Table 3).

## 4. Discussion

In patients with a normal airway, the Miller laryngoscope and the McGrath-MAC VL demonstrated similar effectiveness in terms of C&L and POGO scores when used with the direct lifting technique for epiglottis elevation. Compared to Group M, Group VL had a shorter mean glottic view duration (approx. 8 vs. 10 s), longer intubation duration (approx. 27 vs. 21 s), a lower first-attempt intubation success rate (approx. 67.4% vs. 83.3%), and a higher requirement for external laryngeal pressure (approx. 46.5% vs. 31%); however, no significant differences were observed between the groups. There were no significant differences between the groups in terms of hemodynamic changes related to laryngoscopy.

There are limited studies comparing glottic views using Miller and Macintosh blades in pediatric patients [11,12]. It is generally agreed upon in the literature that laryngoscopy with various devices improves glottic views in different clinical settings, practitioner experiences, and patient groups (pregnant, obese, pediatric, etc.) [5,6,7,8,9,10,11,12,13,14]. A study evaluated the effectiveness of two different methods (direct and indirect) for glottic visualization on the same patient (≥18 years) using AceScopeVL and reported that the indirect epiglottis lifting method was superior in revealing glottic views [12]. Studies have reported that POGO scores were similar in laryngoscopy with Miller and Macintosh blades in patients under two years of age [9]. In newborns, excellent glottic views were obtained with both direct and indirect visualization methods using Miller blades, and POGO scores were higher compared to Macintosh blades in both positions [13]. In adult patients, the Miller laryngoscope provided a full glottic view in 78% of cases, while the Macintosh laryngoscope provided it in 53% (*p* = 0.0014) [11]. In our study, where we aimed to eliminate the difference in experience in evaluating glottic views in adult patients and all intubations were performed by practitioners who had at least 2 years of clinical experience and were experienced in using both laryngoscopes, we attribute the similar C&L and POGO scores in the groups to the appropriate depth of anesthesia and muscle relaxation during laryngoscopy.

The success of intubation is influenced by several factors such as errors in describing and grading laryngeal view, head position, application of cricoid pressure, degree of muscle relaxation, type or size of the laryngoscope blade, and the practical skills of the operator [1,7,8,9,10,11,12,13,15,16,17,18,19,20,21,22]. In patients with a normal airway, the success rate on the first attempt after more than 15 ETI experiences with McGrath-MAC VL was reported to be 87% [17]. Achieving a better glottic view with VL does not always translate to easier tracheal tube placement. In other words, obtaining a POGO view equivalent to a C&L score of 1 (100%) during VL does not guarantee successful intubation [18]. While study results vary, most suggest that intubation duration is longer with VL [9,10,12,13]. A study investigating the impact of the Truview Evo2 laryngoscope and the Miller laryngoscope on hemodynamics and intubation conditions in newborns (*n* = 119) reported that VL was not advantageous compared to direct laryngoscopy in routine practices for newborns due to prolonging intubation duration [19]. In our study, to ensure that the prolonged intubation duration was not due to inexperience, all intubations were performed by practitioners who had at least 2 years of experience and had performed 30 ETIs with both laryngoscopes. Nonetheless, as seen in many other studies, we also obtained a longer intubation duration in Group VL. While we achieved sufficient glottic view for most cases in Group VL, we believe that the difficulty in advancing the endotracheal tube due to the need for better hand-eye coordination played a significant role in prolonging the intubation duration. Considering that the primary goal for all anesthesiologists is to secure the airway as quickly as possible by achieving successful intubation rather than just a successful glottic view, we still consider the use of the Miller laryngoscope to be more advantageous than the McGrath-MAC VL due to higher first-attempt intubation success, better glottic view, and faster intubation, even though there might not be a statistical difference between them.

The maneuver of aligning the oral and pharyngeal axes required to visualize the glottis with the Macintosh laryngoscope induces sympathetic activity by stimulating the supraglottic region. This response is independent of the shape of the laryngoscope blade (straight or curved) and is considered an excessive hemodynamic stress response during direct laryngoscopy [20,21]. Additionally, the force applied during glottic visualization and airway manipulation during intubation have also been reported to be associated with this stress response [21]. With VLs, there is no need to align the oral, pharyngeal, and laryngeal airway axes, and the lifting force required to expose the glottis is reduced, leading to clearer visualization of the airway anatomy and vocal cords [1]. Furthermore, it is assumed that there will be less mechanical stimulation of pharyngeal structures during VL, resulting in a reduction in the hemodynamic response. However, conflicting results have been obtained regarding the hemodynamic response [5,8,20]. Yokose et al. [20] reported that McGrath-MAC VL could reduce the frequency of post-intubation hypertension compared to Macintosh laryngoscopy. Liu et al. [5] reported that McGrath-MAC VL performed by less experienced anesthesiologists led to a reduced increase in systolic blood pressure after intubation. A study compared direct laryngoscopy and McGrath-MAC VL in bariatric surgery in terms of glottic view and hemodynamics and reported that despite prolonging the intubation duration, McGrath-MAC VL provided better oropharyngeal and glottic views without causing hemodynamic changes, and these results did not affect intubation success [6]. In patients with a normal airway, we attribute the similarity in hemodynamics between the two laryngoscopies to a low ASA risk score, appropriate depth of anesthesia, and the practical experience of the operator.

## 5. Limitations

There were some limitations to this study. First, this prospective study could not be conducted blindly because the anesthesia assistants in the study were experienced in using both blades, which limited the validity of the data. Second, the obtained laryngoscopy and glottic view scores do not fully reflect the true population since cases of expected difficult intubations were excluded from the study. Third, glottic views were assessed in different patients with different laryngoscopes. Therefore, a larger sample size is needed to balance inter-individual variability and contribute to homogeneity. Fourth, the duration of the potential hemodynamic response that may occur when VLs are used with the DV method also needs to be investigated. Lastly, the way McGRATH was used may have affected our results because it was used differently than the manufacturer’s recommendation.

## 6. Conclusions

Due to its ability to provide effective intubation conditions, we believe that the McGrath-MAC VL, when used with the indirect view method, can also be utilized in anesthesia practices alongside the DV method.

## Figures and Tables

**Table 1:** Patients’ characteristics and a comparison of Mallampati, C&L-POGO score, glottic vision, and intubation times.

	Group M	Group VL	*p*
Age (years)	38.50 (19–65)	44.00 (18–64)	0.053
Height (cm)	163 (153–184)	165 (143–181)	0.874
Weight (kg)	72 (42–92)	72 (34–98)	0.532
Mallampati	2 (1–2)	2 (1–2)	0.944
C&L score	1 (1–3)	1 (1–3)	0.703
POGO score	80 (20–100)	70 (30–100)	0.099
Glottic vision times (s)	10 (5–50)	8 (2–60)	0.064
Intubation times (s)	20.50 (7–65)	27 (10–80)	0.106

Mann–Whitney U test, Two independent samples *t* test; Median: Minimum–Maximum, Group M: Miller laryngoscope, Group VL: McGrath-MAC VL, C&L: Cormack ve Lehane; POGO: percentage of glottic opening; sec: second.

**Table 2:** Comparison of categorical variables according to groups.

	Group M *n* = 42 (%)	Group VL *n* = 43 (%)	*p*
Gender			
Woman	31 (73.8)	28 (65.1)	0.526
Men	11 (26.2)	15 (34.9)	
External laryngeal compression			
Yes	13 (31)	20 (46.5)	0.212
No	29 (69)	23 (53.5)	
ASA			
1	14 (33.3)	12 (27.9)	0.759
2	28 (66.7)	31 (72.1)	
Number of intubation attempts			
1	35 (83.3)	29 (67.4)	0.148
2	7 (16.7)	14 (32.6)	

Group M: Miller laryngoscope, Group VL: McGrath-MAC VL, ASA: American Society of Anesthesiologists.

**Table 3:** Comparison of hemodynamic responses.

	Group M	Group VL	*p*
MAP			
T1	99.60 ± 11.65	98.81 ± 16.36	0.800
T2	84.02 ± 10.82	86.09 ± 15.19	0.657
T3	104.19 ± 17.13	101.98 ± 18.91	0.358
HR			
T1	83.07 ± 14.03	79.07 ± 12.05	0.162
T2	80.67 ± 14.22	79.16 ± 17.02	0.926
T3	94.69 ± 15.37	91.26 ± 14.22	0.157
SpO_2_			
T1	98.95 ± 1.55	98.93 ± 1.42	0.835
T2	99.55 ± 1.50	99.77 ± 0.57	0.947
T3	99.57 ± 1.06	99.81 ± 0.45	0.465

Mean ± Standard deviation, Group M: Miller laryngoscope, Group VL: McGrath-MAC VL, MAP: Mean arterial pressure, HR: Heart rate, SpO_2_: Peripheral oxygen saturation, T1: before induction; T2: post-induction; T3: during laryngoscopy.

## Data Availability

The data used and/or analyzed during the current study are available from the corresponding author.
